# Supplementary material for: The vaccinia virus DNA polymerase structure provides insights into the mode of processivity factor binding
Source: Nat Commun. 2017 Nov 13;8:1455. doi: 10.1038/s41467-017-01542-z (PMC5682278; doi:10.1038/s41467-017-01542-z)
Supplement: Supplementary file 1 — Supplementary Information [file 41467_2017_1542_MOESM1_ESM.pdf]

Supplementary Table 1 Structural alignment of E9 with different family B polymerases

**Polymerase**	**Kingdom of life**	**State of the enzyme**	**pdb entry**	**RMS deviation of matched residues [Å]**[†]					
				excluding thumb*	thumb	Palm	Finger	N-term	Exo-nuclease
Size (residues in E9)				829	177	249	57	184	258
S. cerevisiae pol δ	eukaryote	ternary	3iay	**3.2**	**2.4**	**1.9**	1.2	8.0	**1.2**
Herpes simplex virus	DNA virus	apo	2gv9	3.8	4.8	3.2	0.9	3.7	2.2
E. coli polymerase II	*bacteria*	ternary	3k57	4.6	6.2	2.0	3.0	**3.6**	2.1
S. cerevisiae pol α	eukaryote	binary	4fxd	4.6	4.5	2.0	**0.7**	10.5	2.8[#]
H. sapiens pol α	eukaryote	ternary<br>apo	4qcl<br>5exr	4.1<br>3.8	5.1<br>4.6	2.1<br>2.1	1.2<br>1.0	7.4<br>8.6	2.0[#]<br>2.6[#]
P. abyssi polymerase	archaea	editing	4flt	4.5	3.0	3.7	0.8	4.6	2.3
T. gorgonarius polymerase	archaea	editing	2xhb	4.8	3.0	3.5	0.7	4.7	2.3
T. gorgonarius polymerase	archaea	binary	2vwj	3.8	3.1	3.1	0.8	5.1	2.3

*Fitting of E9 without the thumb domain.
[†]Domains defined as in Fig. 1 are fitted individually. The "super" command of PyMol has been used for the structural alignment. The lowest values of a column are printed in bold.
[#] Exonuclease is inactive in pol α

Supplementary Table 2 Parameters derived from SAXS measurements

**Structural parameters**	**A20 C-ter**	**E9 exo^{minus}/DNA complex**	**E9**
R_{max}* (nm) [from P(r) function]	7.4	12.5	12.5
R_g† (nm) [from P(r) function]	2.15	3.45	3.83
R_g† (nm) [from Guinier plot]	2.14 ± 0.01	3.47 ± 0.02	3.90 ± 0.02
Porod volume V_p (nm^3)	31	210	202
Molecular mass M_r [from V_p] (kDa) using a specific volume of 1.65 $Å^3Da^{-1}$	19	127	122
Monomeric molecular mass calculated from sequence (kDa)	17	E9 exo^{minus}+ DNA<br>117 + 8 = 125	117

*R_{max}: Maximal dimension of the molecule
†R_g: Radius of gyration

Supplementary Table 3 Oligonucleotide primers used in this study.

**Name**	**Sequence***
E9-578-a-rev	tcttc**GGC**tctattggtactaac
E9-582-a-fw	agaa**GCC**aataatcagctattgc
E9-580-r-rev	t**CT**ttccaatctattggtactaacaac
E9-581-r-fw	**AG**aataaataatcagctattgcttcag
E9-585-a-586-s-rev	caa**GGAAGC**attatttatttcttcttccaatctattg
E9-588-s-589-a-fw	**TCCGCT**aaatatccacctcctagatatattac
E9-exo1-rev	cttcttatcgaagtgaca**CGC**tat**AGC**tagaaataagtacga
E9-exo1-fw	tcgtacttatttcta**GCT**ata**GCG**tgtcacttcgataagaag
A20-414-a-rev†	gattgagacgaactggttaaag
A20-414-a-fw†	**GCT**aatgtcgtcaccgatgtc
A20-410-a-rev	ctggttaaagtttctatatatag
A20-410-a-fw	**GCC**gtctcaatctttaatgtcg
A20-407-a-rev	gtttctatatatagtttcaatactcg
A20-407-a-fw	**GCT**aaccagttcgtctcaatc

*Bases in bold capital letters are different from the wild type sequence

†These primers were used for both A20 C-ter-Phe414Ala and D4/A20-Phe414Ala mutagenesis

Clone:	Length(AA):	Weight(kd):	Sequence alignment: 426	Protein expression:
1	116	15.8	311-VGSAGL{....}KRLFE(...)DIFEAQKIEWHE*	+++
2	122	16.5	305-GKYFSKVGSAGL{....}KRLFE(...)DIFEAQKIEWHE*	+++
**3**	**123**	**16.6**	**304-NGKYFSKVGSAGL{....}KRLFE**(...)DIFEAQKIEWHE*	**+++**
4	124	16.7	303-VNGKYFSKVGSAGL{....}KRLFE(...)DIFEAQKIEWHE*	++
5	129	17.4	298-GRDYYVNGKYFSKVGSAGL{....}KRLFE(...)DIFEAQKIEWHE*	N.A.
6	130	17.5	297-IGRDYYVNGKYFSKVGSAGL{....}KRLFE(...)DIFEAQKIEWHE*	+
7	131	17.6	296-TIGRDYYVNGKYFSKVGSAGL{....}KRLFE(...)DIFEAQKIEWHE*	N.A.
8	145	19.2	282-GTETRGEVIKRIIDTIGRDYYVNGKYFSKVGSAGL{....}KRLFE(...)DIFEAQKIEWHE*	++
9	148	19.5	279-DGNGTETRGEVIKRIIDTIGRDYYVNGKYFSKVGSAGL{....}KRLFE(...)DIFEAQKIEWHE*	++
A20	426	49.2	1...DYDGNGTETRGEVIKRIIDTIGRDYYVNGKYFSKVGSAGL{....}KRLFE	N.A.

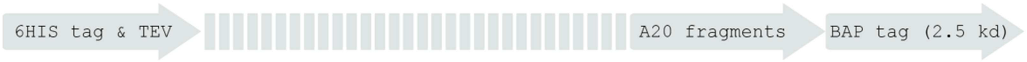

Supplementary Figure 1 Identification of soluble A20 C-terminal fragments generated with the ESPRIT method. The amino acid sequence of the nine independently selected fragments of A20 are shown. The length of each fragment is indicated as well as the corresponding molecular weight taking into account the presence of the BAP-tag. The level of recombinant protein expression (in bacteria) is specified when tested. Clone 3 (depicted in bold) is the construct used throughout our study and is named A20 C-ter. A schematic view of the constructs is shown below.

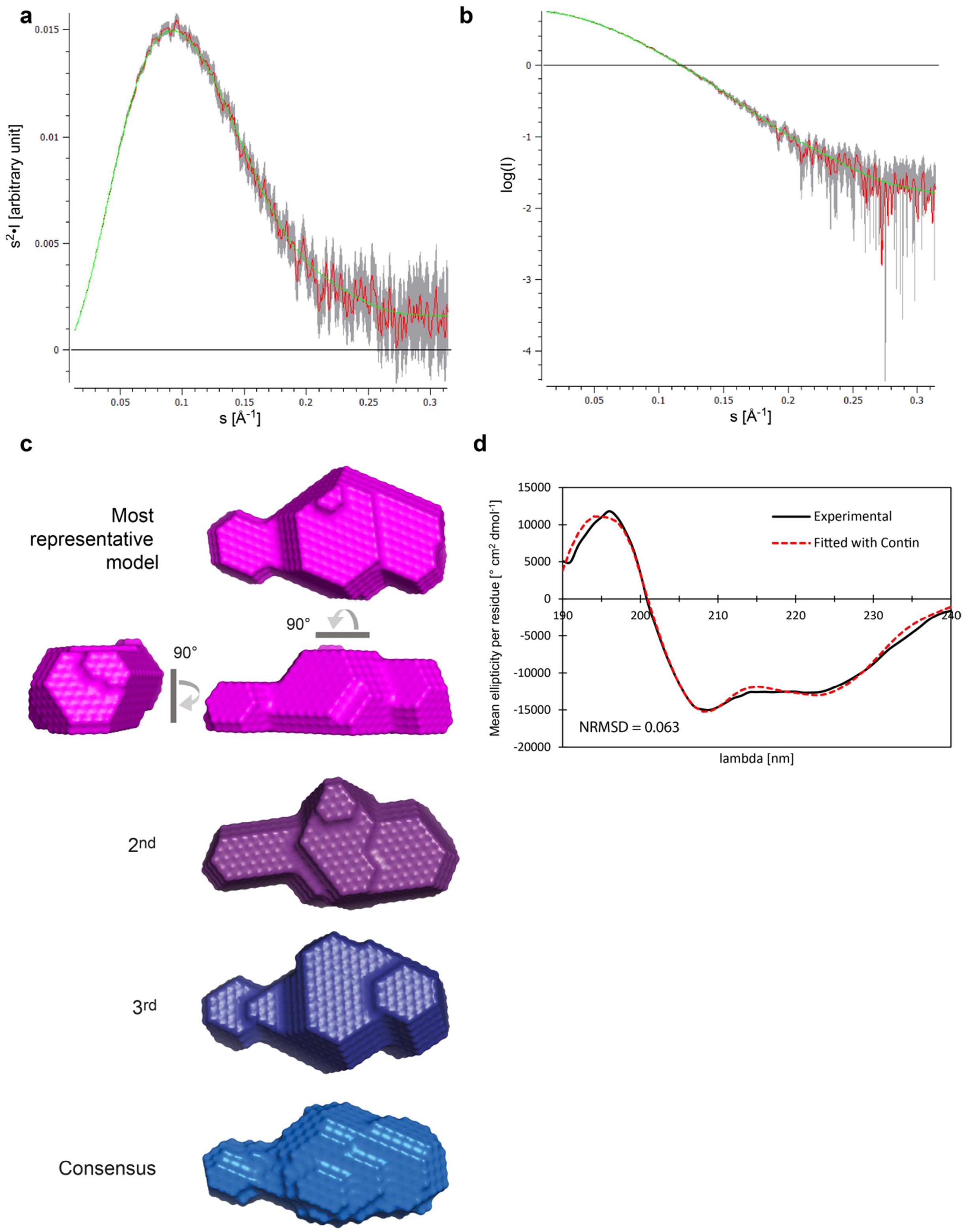

Supplementary Figure 2 Characterization of A20 C-ter. Panel a-c: analysis of SAXS data. **(a)** Kratky plot. **(b)** Fit of the experimental scattering curve with the calculated curve (green line) of the most representative *ab initio* model shown in panel c. **(c)** Views of the most representative model as well as the 2nd, 3rd and consensus models built with DAMMIF. (**d**) Circular dichroism spectrum of A20 C-ter showing both the experimental data (in black) and the curve fitted with Contin (in red).

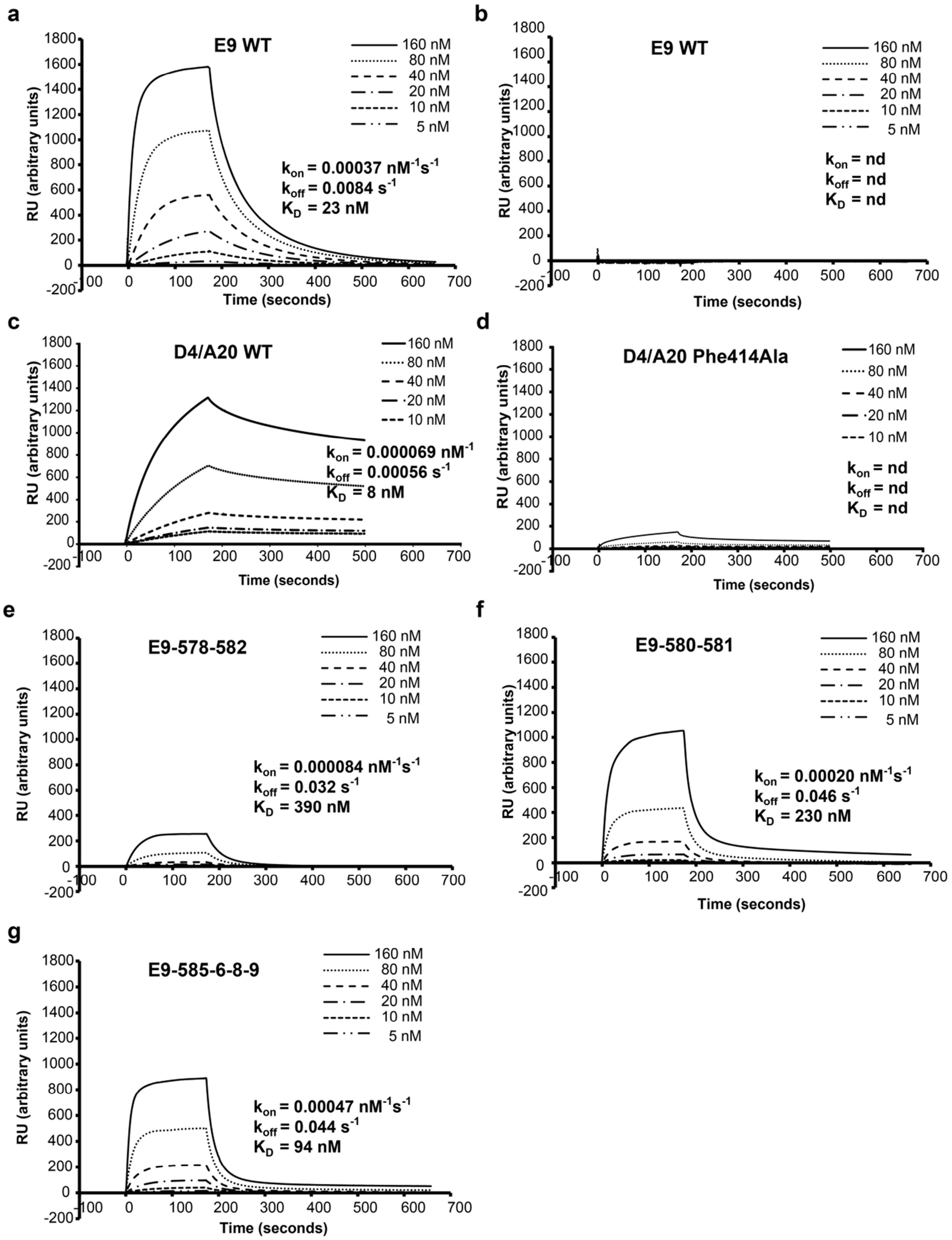

Supplementary Figure 3 Quantitative analysis of E9 interaction with A20 C-ter or full-length D4/A20 using surface plasmon resonance. Curves plot resonance units (RU) as a function of time. BAP-tagged A20 C-ter **(a)** or Phe414Ala mutant **(b)** were immobilized on streptavidin-coated sensorchips. Binding curves for E9 WT on A20 C-ter **(a)**, or A20 C-ter-Phe414Ala **(b).** In **(c)** and **(d),** E9 WT was immobilized and full-length D4/A20 WT **(c)** or the D4/A20 Phe414Ala mutant (**d**) were injected. For the mutant, the dissociation phase shows strong bi-exponential behavior which precludes the determination of the K_D. In **(e)**, **(f)** and **(g)**, BAP-tagged A20 C-ter was coated on sensorchips. Binding curves for E9-578-582 **(e)**, E9-580-581 **(f)** and E9-585-6-8-9 **(g)**.

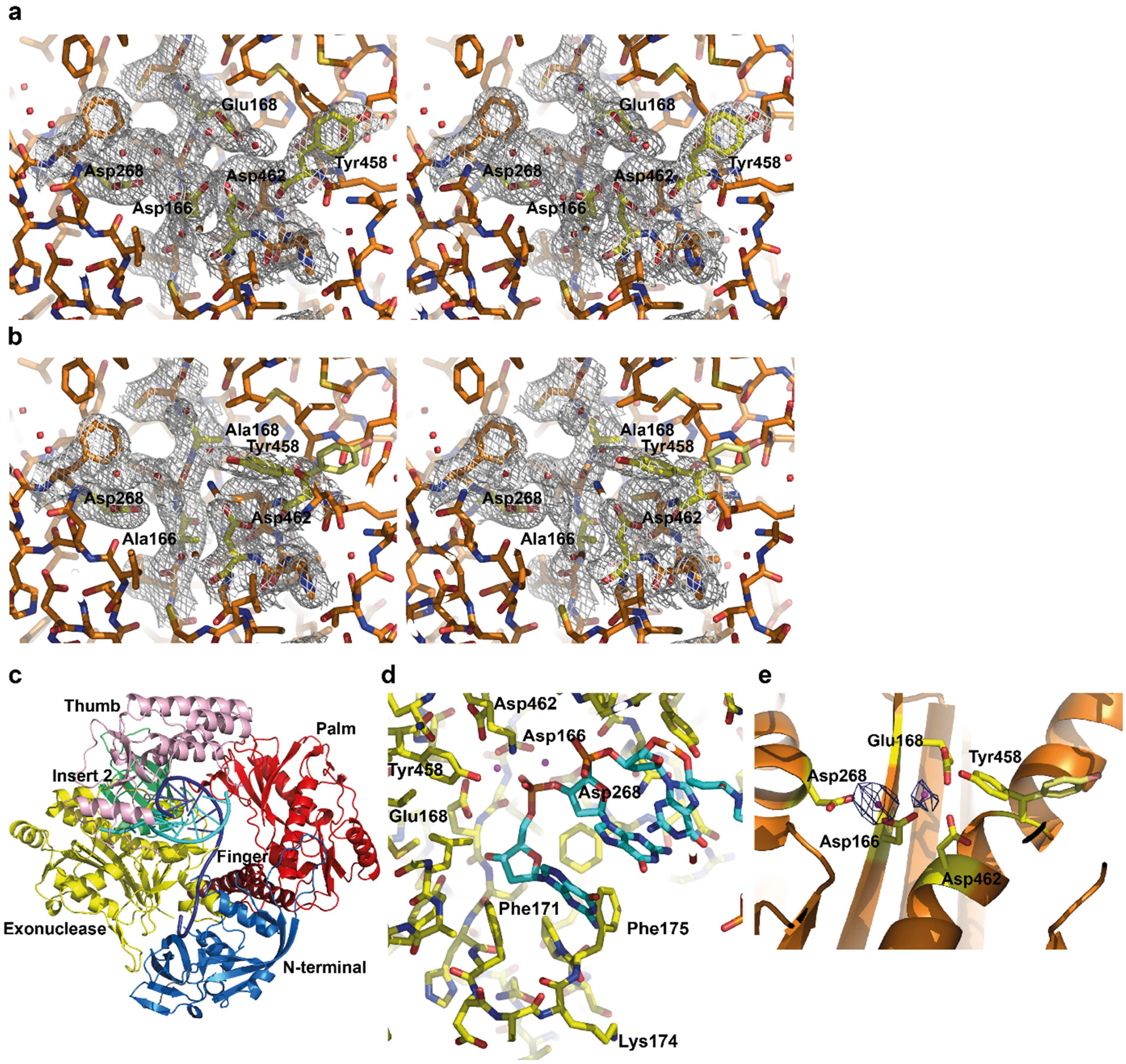

Supplementary Figure 4 The editing mode of E9. **(a)** Stereo view of the E9 WT exonuclease site. Weighted 2Fo-Fc electron density is contoured at 1 σ level in the vicinity of the active site residues. Water molecules are shown as red spheres. Tyr458 has alternate conformations where the one pointing towards the active site has an occupancy of 0.7. Active site residues are labeled. **(b)** Corresponding stereo view of the E9 exo^{minus} mutant (Asp166Ala, Glu168Ala) based on data in Table 1. Note the absence of the side chains of the mutated residues. **(c)** A model of E9 in editing mode built from PDB entry 2xhb of the archaeal polymerase from *T. gorgonarius* in complex with hypoxanthine-containing DNA. Domains of E9 have been positioned individually by structural alignment; the DNA shown is the one from PDB entry 2xhb. **(d)** Magnified representation of the exonuclease active site of the model in panel c. Catalytic residues and residues involved in resistance mutations Phe171Ser and ΔLys174 are labeled. Mn^{2+} ions (magenta spheres) have been placed according to the Mn^{2+}-bound E9 structure shown in panel e (see data in Table 1) **(e)** Mn^{2+}-bound E9. The anomalous electron density is contoured at 4 σ and shows the Mn^{2+} atoms (magenta spheres).

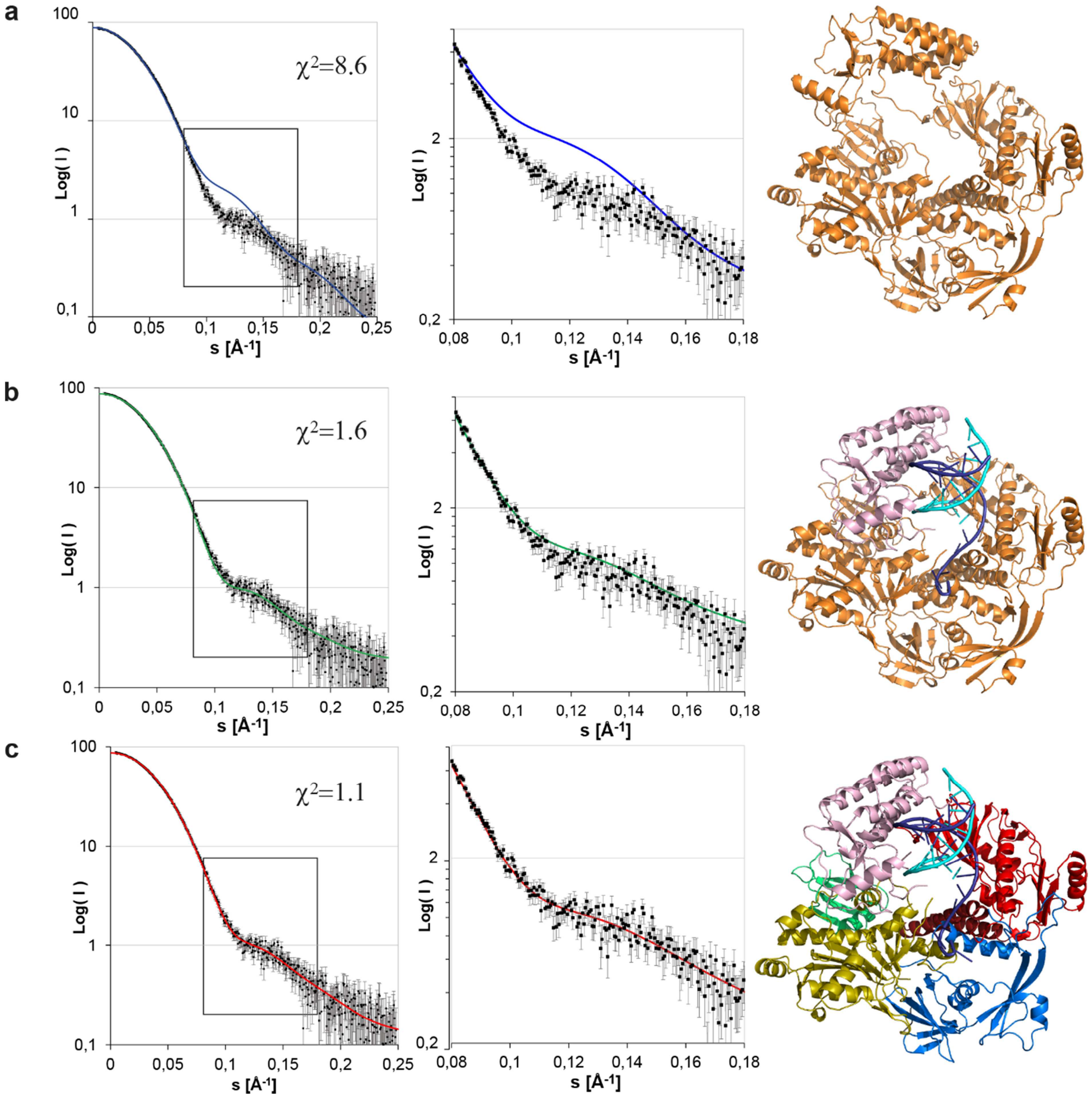

Supplementary Figure 5 Agreements of scattering curves calculated from models of E9 with SAXS data collected on E9 exo^{minus} with a bound 29-mer DNA oligonucleotide hairpin structure. Theoretical scattering curves calculated from the model structures (depicted on the right) and experimental SAXS curves (black dots with error bars) are shown together with the χ^2 of the agreement between model and experimental data. The middle panel shows a zoom in the 0.08 - 0.18 Å^{-1} range from the graph on the left. **(a)** The crystal structure of apo E9 is used for the theoretical scattering curve plotted as a blue line. **(b)** A model of E9 in elongation mode based on the ternary complex of yeast polymerase δ (PDB 3iay) has been used (green line). Domain movements have been modeled with 2 domains, the thumb domain and the remaining structure. **(c)** The model of E9 based on the superposition of individual domains onto yeast polymerase δ has been used to calculate the scattering curve. Exonuclease domain and insert 2 have been handled as one rigid body as insert 2 does not have an equivalent in polymerase δ and residues 497-523 of the N-terminal domain have been fitted together with the palm domain.

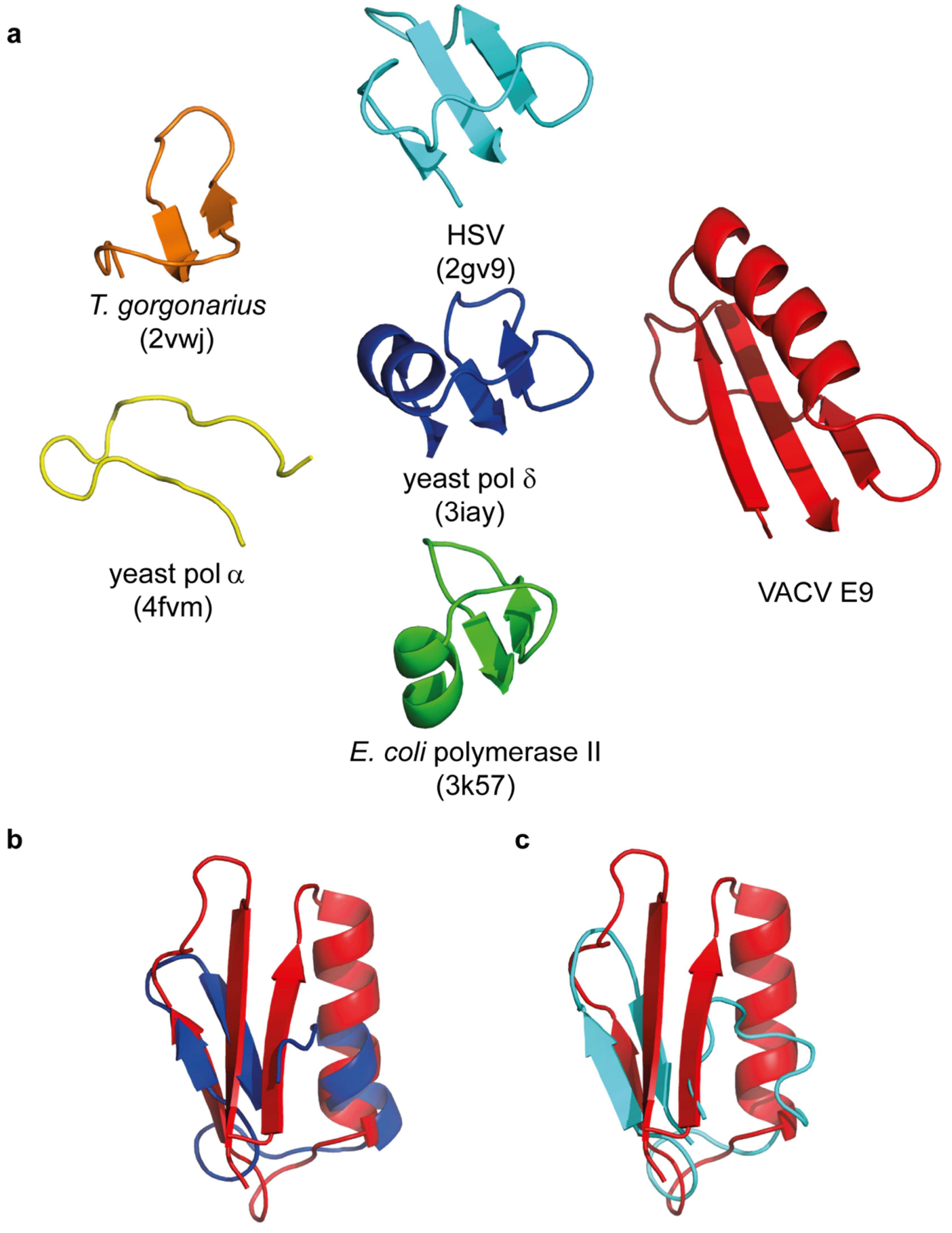

Supplementary Figure 6 Evolution of the small domain (residues 567 to 617) carrying E9 insert 3. **(a)** The insert 3 domain and corresponding residues of other polymerases are shown. Polymerase structures have been superposed and the same orientation is used throughout the panel. **(b)** Structural alignment of the insert 3 domain of E9 with that of yeast polymerase δ and **(c)** HSV DNA polymerase. Structural alignments used the “super” command of PyMol.

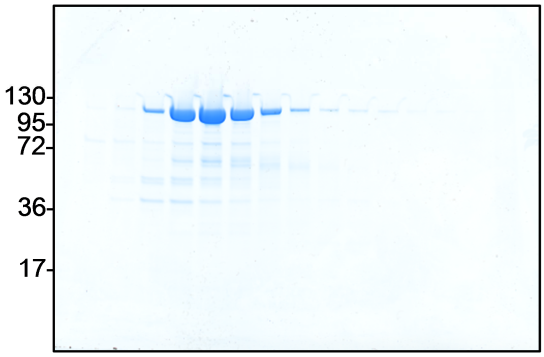

Fig 2b : E9

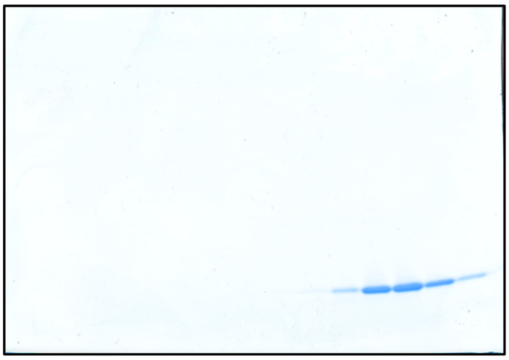

Fig 2b : A20 C-ter

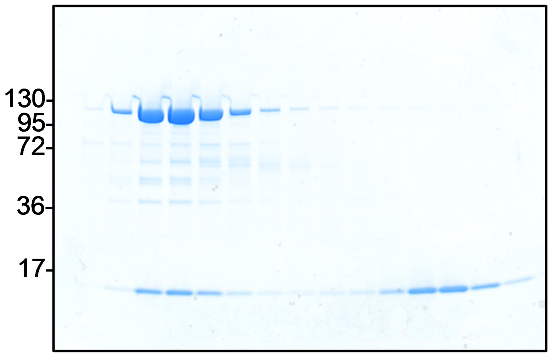

Fig 2b :E9 + A20 C-ter

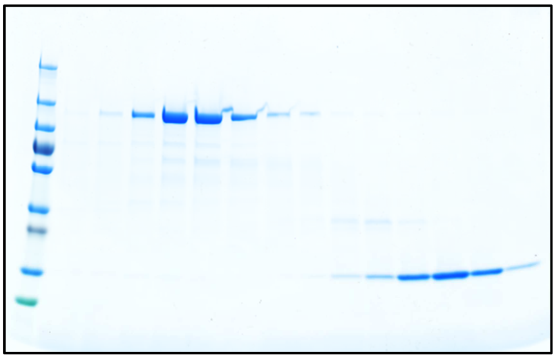

Fig 2e :E9 + A20 C-ter-Phe414Ala

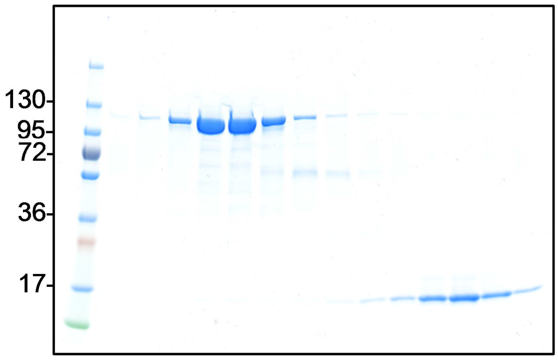

Fig 4b : E9-578-582

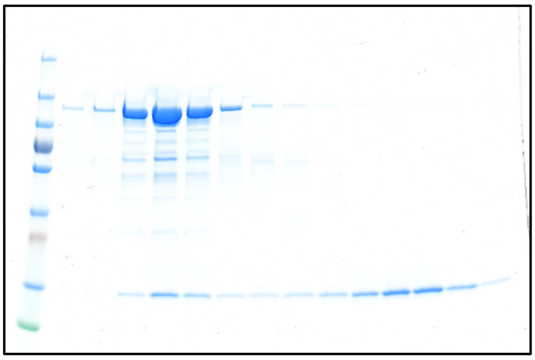

Fig 4d : E9-580-581

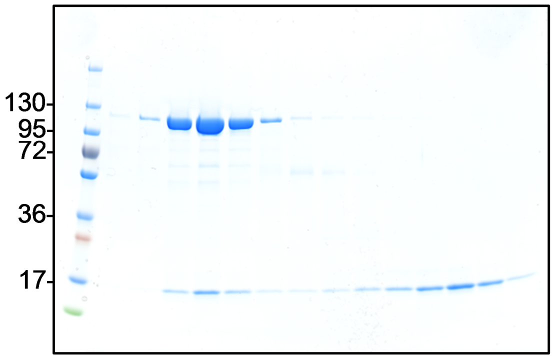

Fig 4f : E9-585-6-8-9

Supplementary Figure 7 Uncropped images of the gels shown in Figs. 2 and 4.
